# Survey of potential receptivity to robotic-assisted exercise coaching in a diverse sample of smokers and nonsmokers

**DOI:** 10.1371/journal.pone.0197090

**Published:** 2018-05-10

**Authors:** Christi Patten, James Levine, Ioannis Pavlidis, Joyce Balls-Berry, Arya Shah, Christine Hughes, Tabetha Brockman, Miguel Valdez Soto, Daniel Witt, Gabriel Koepp, Pamela Sinicrope, Jamie Richards

**Affiliations:** 1 Department of Psychology and Psychiatry, Mayo Clinic, Rochester, Minnesota, United States of America; 2 Department of Obesity Solutions, Mayo Clinic, Scottsdale, Arizona, United States of America; 3 Obesity Solutions, Arizona State University, Tempe, Arizona, United States of America; 4 Department of Computer Science, University of Houston, Houston, Texas, United States of America; 5 Department of Health Sciences Research, Mayo Clinic, Rochester, Minnesota, United States of America; 6 Center for Clinical and Translational Science, Office for Community Engagement in Research, Mayo Clinic, Rochester, Minnesota, United States of America; 7 Mayo Clinic School of Medicine, Mayo Clinic, Rochester, Minnesota, United States of America; 8 Rochester Area Family YMCA, Rochester, Minnesota, United States of America; Tokai University, JAPAN

## Abstract

A prior project found that an intensive (12 weeks, thrice weekly sessions) in-person, supervised, exercise coaching intervention was effective for smoking cessation among depressed women smokers. However, the sample was 90% White and of high socioeconomic status, and the intensity of the intervention limits its reach. One approach to intervention scalability is to deliver the supervised exercise coaching using a robotic human exercise trainer. This is done in real time via an iPad tablet placed on a mobile robotic wheel base and controlled remotely by an iOS device or computer. As an initial step, this preliminary study surveyed potential receptivity to a robotic-assisted exercise coaching intervention among 100 adults recruited in two community settings, and explored the association of technology acceptance scores with smoking status and other demographics. Participants watched a brief demonstration of the robot-delivered exercise coaching and completed a 19-item survey assessing socio-demographics and technology receptivity measured by the 8-item Technology Acceptance Scale (TAS). Open-ended written feedback was obtained, and content analysis was used to derive themes from these data. Respondents were: 40% female, 56% unemployed, 41% racial minority, 38% current smoker, and 58% depression history. Mean total TAS score was 34.0 (SD = 5.5) of possible 40, indicating overall very good receptivity to the robotic-assisted exercise intervention concept. Racial minorities and unemployed participants reported greater technology acceptance than White (p = 0.015) and employed (p<0.001) respondents. No association was detected between the TAS score and smoking status, depression, gender or age groups. Qualitative feedback indicated the robot was perceived as a novel, motivating, way to increase intervention reach and accessibility, and the wave of the future. Robotic technology has potential applicability for exercise coaching in a broad range of populations, including depressed smokers. Our next step will be to conduct a pilot trial to assess acceptability and potential efficacy of the robotic-assisted exercise coaching intervention for smoking cessation.

## Introduction

Smokers with current depression are a tobacco use disparity group [1–3] and few treatment studies have targeted this population [4–9]. Supervised exercise has potential applicability as a smoking cessation treatment for depressed smokers. Our recent pilot study targeted women with moderate-severe depressive symptoms and evaluated the efficacy of a 12 week, thrice weekly, supervised, vigorous intensity exercise intervention for smoking cessation [10]. All participants received behavioral counseling and nicotine patch therapy for quitting smoking. Thirty women were randomized to the exercise intervention delivered in a community YMCA setting (n = 15) or health education contact control condition (n = 15); both were delivered by research wellness coaches. Exercise session attendance was high. At week 12 (end-of-treatment) the exercise condition was associated with significantly higher biochemically verified smoking abstinence rates (73% [11/15]) compared to controls (33% [5/15]); *p* = 0.028. No statistically significant differences between groups were detected at six-month follow-up. Thus, vigorous intensity supervised exercise is feasible, and, as our data suggest, enhances short-term smoking cessation, among depressed female smokers. The study was limited with respect to sample characteristics: 90% White and high socioeconomic status.

**Fig 1:**
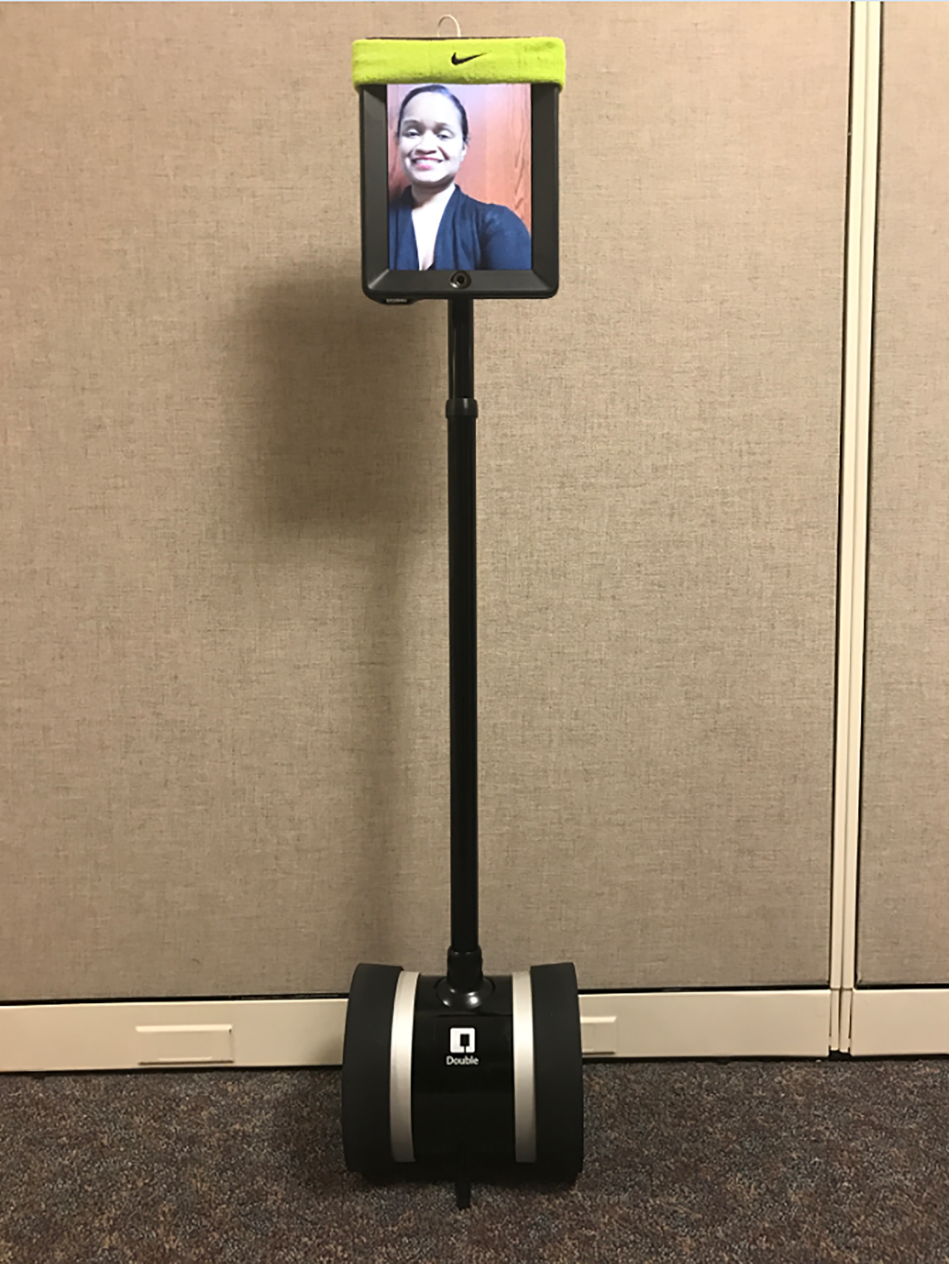
Robotic human exercise trainer technology.

As such limitations exemplify, a key challenge for future studies is to discover innovative and cost effective strategies to bolster long-term adherence among a diverse range of participants, including those living in remote impoverished communities, while additionally considering that supervised exercise is associated with better outcomes in studies of both depression [11] and smoking cessation [12–15] (see also Ussher et al. [16] for review). Studies examining the use of home-based exercise counseling (non-supervised) among depressed smokers revealed that such methods were not effective for promoting exercise adherence or smoking cessation, presumably due to the lack of supervision and support [4, 5]. A potential new approach to intervention scalability in diverse community settings is to deliver exercise coaching to smokers through a robotic system human interface (i.e., robotic human trainer; see Fig 1). This can be done in real time via an iPad tablet placed on a mobile, robotic wheel base controlled remotely by an iOS device or computer. The technology was originally developed for secondary education to reach remote learners.

The initial prototype is a live exercise coach interacting with participants in real time through the robotic interface. The supervised intervention delivered is identical to an in-person coach except that the coach will interact with the participant remotely through the robotic telepresence interface (Fig 1). The coach will perform the same tasks but instead of being physically present with the participant, the coach will function remotely from any location. The robotic interface can move around the participant, not only to examine how the participant performs an exercise but to facially interact with him/her. A robotic-assisted trainer could thus achieve the same functions as an in-person coach including supervision, instruction, accountability, and reinforcement.

One alternative is the use of digital health coaching through internet and mobile phone applications [17, 18]. For example, in depression treatment, support provided by a virtual coach (i.e., embodied conversational agent) can facilitate participant engagement and self-disclosure due to its perceived anonymity and non-judgmental nature [19]. However, these technologies emulate, but notably do not include, the support of a live coach. At this early stage of the research, the current prototype bridges the gap between human and embodied support. If successful, future research could scale up both the robotic device to enhance portability (e.g., mobile phone app) as well as its interface to a semi- or fully-automated system. The significance of robotic human exercise coaching is its potential scalability and its reach to large populations at a relatively low cost [20, 21].

In this preliminary survey study, we evaluated receptivity to the robotic-assisted exercise coaching intervention concept in a racially and economically diverse convenience sample of 100 adults. Evaluation of potential acceptance of an intervention model is a recommended first step in developing new technology-based behavioral addictions treatments [22]. We used the Technology Acceptance Model [23], frequently applied to evaluate technology acceptance [22, 24, 25]. Two aspects of this model are: (1) perceived usefulness or the degree to which a person believes that using a particular system would enhance his or her personal health objectives and (2) perceived ease of use which refers to the degree to which a person believes the system would be free of effort. These acceptance indicators have been shown to predict actual adoption of new technologies [23, 25]. We explored differences on potential acceptance of the robotic human trainer technology by smoking status and other demographic characteristics.

## Methods

The study was approved by the Mayo Institutional Review Board.

### Participants

Data were collected in January, 2017 and analyzed in 2017. A convenience sample of participants was recruited at two community settings serving low income populations, the YMCA and the public library, in southeastern, MN. The targeted sample size was 100 participants. At the YMCA, a booth was set up for recruitment at the fitness center entrance. At the library, information about the study was posted on the library web calendar. Flyers were posted in both locations. Recruitment advertisements described the project as a study to obtain feedback on using a “robotic human exercise trainer” for promoting exercise. Screening was done by study staff in person to assess the eligibility criteria: (1) 18 years of age or older and (2) provided oral consent to participate. All participants screened were eligible and elected to participate. Participants were provided with a $25 Visa gift card as a thank you for their time.

Respondents were: 40% female sex (100% concordant with reported gender identity), 41% racial minority (31 participants were Black, 8 American Indian/Alaska Native and 2 Asian), and 56% were unemployed. Thirty percent of respondents were aged 18–29 years, 42% were 30–49 and 28% were aged 50 or older. Thirty-eight percent were a current smoker, 58% reported a depression history and 66% did not meet ACSM recommended guidelines for physical activity. Mean days/week of physical activity was 3.7 (SD = 2.0, range 0–7).

### Study design

Cross-sectional, mixed methods survey design.

### Materials

The current prototype required set up in a community setting with Internet and wireless (Wi-Fi) availability. Research staff set up the robotic interface prior to each demonstration. The prototype demonstrated to participants was a live exercise coach interacting with participants in real time through a robotic telepresence interface (see Fig 1). An iPad tablet is placed on a mobile, robotic wheel base and is controlled by the coach from another location by using an iOS device or computer. The entire device measures 5 feet, 1 inch in length.

The current prototype for delivery of a supervised exercise intervention is identical to an in-person coach except that the coach can interact with the participant remotely through the robotic telepresence interface. The coach can perform the same tasks but instead of being physically present with the participant, the coach functions remotely from any location. The robotic interface can move around the participant, not only to examine how the participant performs an exercise but to facially interact with him/her. A robotic-assisted trainer can thus achieve the same functions as an in-person coach including supervision, instruction, accountability, and reinforcement. The technology is different than a web-assisted video or skype phone application because the robot device can accompany and move with and around the participant during exercise, and the participant remains hands-free. Thus, the device could be practical with groups of individuals exercising at the same time.

### Procedures

Research staff facilitated the robot demonstration and administered the survey at two community settings. Staff members from the community settings were also present to facilitate the research. Participants were read the oral consent form. Study staff then briefly described results from the previous supervised exercise study, that the intervention was delivered by an in-person coach at a community YMCA setting, and comprised 12 weeks of thrice weekly sessions [10]. Study staff explained that our interest was in evaluating potential receptivity to the robotic-assisted technology for delivery of the individual-based, supervised exercise coaching intervention to smokers with depression. It was explained to participants that the robot would be set up and accessed by participants in a community setting.

Demonstration of the robotic human trainer was provided to participants in a private room outside of the fitness center (YMCA) or in a group study room or wellness corner (library). The participant watched the demonstration alone or with a group of other participants. The demonstration lasted about 3–5 minutes.

The coach (shown in Fig 1) was a Black female and was consistent for all participants. The coach gave written consent (as outlined in PLOS consent form) to publish these details. The coach first remotely moved the robotic device into the room with participant(s). The researchers explained that the coach was controlling the device from another location. Next, the coach introduced herself and then used a script adapted from our effective exercise coaching intervention for smoking cessation [10] as a mock session:

- *Hi my name is _____ (coach)*. *It’s nice to see you (all of you)!*
- *As a robotic trainer*, *my role is to help people to exercise more but I also focus my time on them and how they are doing*. *My role is to support and encourage people even while they are exercising*. *I try to bring positive energy to the exercise sessions and help them see all of the important progress they are making*, *even when it is tough going at times*.
- *Let’s talk about some of the benefits of exercise for* *you*. *What is important to you?*
- That’s good! Anything else you have noticed?
- *(if not already mentioned)*: *Some people also say that exercise gives you*: *more energy*, b*etter sleep*, *a sense of accomplishment*, d*ecreased stress*, *a way to manage weight*, *a way to stop cravings for sugar*.
- *Great*, *keep these benefits in mind* *every time* *you exercise or when you are starting to think about beginning to exercise*. *That’s all for today*.
- *Thanks for stopping by*, *and I look forward to seeing you again*.

After watching the demonstration, participants were instructed “We would like your feedback on the robotic trainer. We want to know what you think about using the robotic trainer to assist people to exercise.” Participants individually completed a one-time, 2-page, 19-item self-administered survey assessing socio-demographics and acceptance of the robotic human trainer, which took about 5–10 minutes to complete. The survey did not request any identifying personal information. Participants were asked to put the survey in a collection box. After completing the survey participants received a gift card, and at that time, their study involvement was completed.

### Measures

A 19-item survey was developed by the research team.

#### Socio-demographics

Participant characteristics were assessed, including: age group (18–29, 30–49, 50+ years), sex, gender, race, and employment status. Individuals were asked “What is your smoking status?” with response options: never smoked, former smoker or current smoker. Current or past history of depression was assessed using the question “Have you ever experienced depression now or in the past?” with response options Yes or No. Individuals were also asked to indicate how many days (0–7) they engaged in moderate intensity activity “hard enough to break a sweat” on a typical week with examples of activities including running, walking and biking. Meeting the American College of Sports Medicine (ACSM) [26] recommended guidelines for physical activity was defined as at least 5 days of moderate activity.

#### Technology Acceptance Scale (TAS)

Potential receptivity to the robotic human trainer was assessed through participant responses to eight items adapted from the TAS, a valid and reliable measure of technology acceptance [23]. The items were based on: (1) perceived usefulness and (2) perceived ease of use. Each item was rated on a five point Likert scale ranging from 1 (strongly disagree) to 5 (strongly agree). Two of the items are reverse scored. The total TAS score could range from 5–40 with higher scores indicating greater potential receptivity to the technology. Internal consistency reliability of the scale was high (α = 0.85) for this sample.

#### Open-ended feedback

Participants were asked to write in responses to two open-ended questions that assessed general reactions to robots and the use of new technologies for health promotion. These questions were “Tell us what you think about using technology to help you and others to be healthy,” and “What do you think about robots?”

### Data analysis

#### Quantitative data

The study was designed to have 90% power for detecting a median effect (0.65) of smoking status and other socio-demographic categorical variables on the mean difference in Technology Acceptance Scale total scores (SD = 1.0) [27]. Socio-demographic characteristics and TAS item and total scores were summarized for the overall sample using descriptive statistics. Two-sample t-tests and analysis of variance were used to examine differences in mean TAS scores based on smoking status and other demographic characteristics. P values of <0.050 denoted statistical significance.

#### Qualitative analysis

All survey participants responded anonymously to both open-ended written questions. Data saturation was reached with 100 respondents. A word document of 200 responses (100 per question) was generated and combined for analysis because when asked about perceptions of using technology for improving health many participants referred to the robot. Content analysis [28] was the theoretical framework that was used to generate response themes from the data. Two co-authors (CP and AS) coded all responses independently with inter-rater agreement of 88%. Coders were both female, one being a PhD Professor and one being a medical student, with both having experience in qualitative methods and analysis. Discrepancies in coding were resolved through discussion with a third author (TB) until consensus was reached. We did not perform member checking or obtain feedback from participants on results as the survey was self-administered and completed anonymously.

## Results

### Technology Acceptance Scale scores

**Table 1** shows mean item and total scores on the TAS. Mean item scores ranged from 3.91 to 4.52 out of a possible 5.0. The mean total TAS score was 34.0 (SD = 5.5), indicating overall very good receptivity to the robot.

**Table 1:** Technology Acceptance Scale item and total scores (N = 100)^a^.

Item	Mean ± SD^b^
1. The robot trainer was clear and understandable.	4.52 ± 0.87
2. I would find it easy to ask the robot trainer something.	4.42 ± 0.93
3. It would take a lot of effort to interact with the robot trainer.	3.91 ± 1.28
4. I would feel confident interacting with the robot trainer.	4.31 ± 0.96
5. I would find it easy to interact with the robot trainer.	4.30 ± 0.86
6. The robot trainer could help to encourage me to exercise.	4.18 ± 1.03
7. I would find it frustrating to interact with the robot trainer.	4.19 ± 1.03
8. The robot trainer could be helpful for me when exercising.	4.18 ± 0.95
***Total Score***	34.01 ± 5.54
Range	16–40

^a^Items were rated on a 5 point Likert type scale ranging from 1 (strongly disagree) to 5 (strongly agree). Items 3 and 7 were reversed scored such that a higher score indicated less effort (item #3) or less frustration (item #7). The total score has a possible range of 5–40 with a higher score indicating greater acceptability of the robot technology.

^b^Each of the 8 items had an observed range of 1–5.

### Association of Technology Acceptance Scale scores and demographic characteristics

The mean total TAS score was associated with employment status such that those who were unemployed indicated greater technology acceptance than employed respondents (mean ± SD = 35.91 ± 4.60 vs. 31.59 ± 5.74; *t* = 4.180, *df* = 98, *p*<0.001). The TAS score was also significantly associated with race (*t* = 2.468, *df* = 98, *p* = 0.015), with the mean score higher among non-White respondents than for White respondents (35.61 ± 5.43 vs. 32.90 ± 5.39).

Mean TAS total scores were not associated with gender (p = 0.95), age (p = 0.99), smoking status (p = 0.17), history of depression (p = 0.14), or meeting ACSM guidelines for physical activity (p = 0.44).

Because of our particular interest in using the robotic technology for depressed smokers, we compared individuals who were current smokers who self-reported a depression history (n = 30) to all remaining respondents (n = 70). Mean TAS scores were higher among smokers with depression history but not significantly so (35. 01 ± 5.80 vs. 33.54 ± 5.40; p = 0.20).

### Qualitative feedback on robots and other technology for promoting health

Of the 200 written responses, 185 were coded as positive comments, 10 were negative and 5 indicated uncertainty. Four major themes emerged from these data, described below with illustrative quotes.

#### 1. The use of robots and technology is a novel idea

The first theme was that technology was a **novel** idea. Use of technology was described as risky (in a good way), creative, clever, and cool. Respondents described the use of robots as novel/unique, intriguing, exciting, fun, wonderful, good to have, needed, and a good way to help people.

• *It’s another avenue to help people*. *Some people may respond more positive to this over a real person because it’s less intimidating*. *(#80*, *White male)*

• *It’s a very creative use of technology*. *It’s a crazy good idea! (#46*, *Black female)*

• *I think it would be fun…new experience*. *(#39*, *Black female)*

#### 2. Robots and other technology can increase intervention reach, accessibility and cost-effectiveness

Technology was viewed as a way to increase the **reach** of exercise interventions to people wherever and whoever they are, and at lower cost. Examples of groups of people technology could reach were overweight, disabled, those with chronic disease, and those who preferred to exercise at home.

• *We are in the age of technology so I believe the robot trainer is a wonderful idea*. *It will be good so people can be somewhere else and still be right there to assist*. *(#15*, *White male)*

• *I think it’s a great idea to keep using and finding ways to reach others who aren’t physically here*. *(#17*, *White male)*

• *It can be used in a more private setting too*. *(#4*, *White male)*

• *It’s a great idea and may be cost-effective*. *(#33*, *White male)*

• *I think a robot would be helpful if it costs me less and motivates me while I am exercising*. *(#70*, *Asian male)*

• *Convenient (#30*, *White male)*

• *It would be helpful to people who can’t get around*. *(#50*, *White male)*

#### 3. Robots could help motivate people to exercise

Technology was seen as a potentially helpful way to **motivate** the respondent or others to exercise.

• *I’m shocked-this could really provide the missing incentive I need to get on track*. *(#47*, *White female)*

• *It’s an excellent concept of motivation*. *(#12*, *Black male)*

• *It’s a way to have someone encourage you if there is no one to do so*. *(#68*, *White female)*

#### 4. Technology has an increasing role in society -with some limitations

It was acknowledged that technology is “now,” the **wave of the future**, and may even replace humans. Technology was viewed as a new way to educate and help people learn how to exercise.

• *I think since today’s age people use technology*. *I think the best way for someone to learn off of technology*. *(#44*, *American Indian/Alaska Native male*, *never smoked)*

• *Learning about things through technology is very cool*. *(#43*, *American Indian/Alaska Native male)*

However, the 15 respondents who were not certain or had a negative response about robots or technology expressed concerns about privacy, technology replacing human interaction, or even taking jobs away from humans. Moreover, it was expressed that there was a learning curve to using technology; the robots were only as good as the technology or programming. In addition, the motivation and personality of the robotic human interface was a key factor to its acceptability.

• *Good technology and would need to get used to it over time*. *(#85*, *White male)*

• *It’s a tool and the effectiveness of the tool is all about the interest and motivation of the user*. *I think it will be hard to capture the quality of face-to-face interaction*. *(#3*, *White male)*

• *They (robots) only know what they are programmed to know*. *(#81*, *White female)*

• *The robot is an extension of the trainer’s personality*. *If the trainer works well with people and is knowledgeable that would work best*. *(#93*, *Native Hawaiian/Pacific Islander female)*

## Discussion

Previously described exercise interventions for smoking cessation have been conducted by exercise coaches and trainers using phone counseling and face-to-face delivery formats [16]. However, the use of robotic-assisted technology to provide supervised exercise coaching has not been explored in the context of helping to promote smoking cessation. This study assessed likely acceptance among potential users which is consistent with the recommended first step for creating or adapting new technologies for behavioral addictions treatments [22]. Overall, there was high potential receptivity to the robotic-assisted exercise intervention concept in a diverse sample of smokers and nonsmokers. The finding that racial minorities and people who were unemployed reported greater potential acceptance of the robotic technology is encouraging for ultimate intervention scalability and reach to underserved, impoverished communities who have available public libraries and other community settings with web access. Moreover, results indicated no differences in receptivity between smokers and nonsmokers or other demographic characteristics (depression history, gender, age), suggesting the possibility of broad applicability of the technology.

This preliminary study has several strengths including, the solid conceptual framework and measure of technology acceptance, recruitment in two community settings serving low resourced populations, and inclusion of a diverse sample. Using mixed methods, the qualitative findings complemented the quantitative survey data, providing insights into the overall high potential receptivity to the robotic-assisted exercise coaching.

Weaknesses of this study are the relatively small sample size inherent in a first phase study of technology development and use of a convenience sample limiting generalizability. That noted the study was adequately powered to address our hypotheses. In addition, our assessments of tobacco use, depression and physical activity were limited. The survey was kept brief to reduce respondent burden as the main goal was to assess likely receptivity to the robot as this has not been done before. Our measures of depression and smoking status were developed for this study but similar to other validated single item measures [29, 30]. Another limitation is that we assessed potential receptivity to a brief, mock introductory coaching session and not exposure to the actual intervention. Although the intervention was introduced in the context of a smoking cessation intervention, the coaching script focused on exercise and did not include content that addressed quitting smoking. Our next step will be to conduct a pilot trial to evaluate acceptability and potential efficacy of supervised, robotic-assisted exercise coaching intervention for smoking cessation in a diverse sample of depressed male and female smokers. The current study, along with the next evaluation phase (pilot trial), could broadly contribute to the field of digital technologies for behavior change. Our current prototype bridges human and embodied support, but future applications could include a semi-or fully automated interface to increase scalability and cost-efficiency.

Noting the prevalence of smoking and depression in low-income people, robotic trainers are one potentially valuable tool for helping to improve physical activity in people living in impoverished conditions who are especially prone to sedentariness [31–34]. Furthermore, the potential receptivity of the robotic technology especially amongst people from underserved populations heightens the importance of examining this approach in other conditions linked to poverty and health disparity such as obesity and diabetes [35]. One challenge is individual access to and use of the Internet [36]. However, the current prototype required set up in a community setting (e.g., YMCA) with Internet and Wi-Fi availability, potentially enhancing reach to low income adults who individually may have limited individual Internet access or use [37]. Nonetheless, use of the robotic telepresence would require some initial training and familiarization (e.g., powering the robot on and off, initial set up, and learning how to control and move the device) for staff less familiar with computers and/or digital applications.

A recent mixed methods study found that preferred use of technology for exercise coaching among adults with chronic obstructive pulmonary disease included both dialogue support and primary task support [38]; these features can be delivered by a human robotic exercise trainer. Our findings indicate the urgent need to explore using robotic trainers to deliver exercise interventions to a wide range of populations, not only depressed smokers.

## Supporting information

S1 File Excel data set. (XLS)
